# Economic evaluation of biomarker-based surveillance for Hepatocellular Carcinoma in Thai patients with Compensated Liver Cirrhosis

**DOI:** 10.1371/journal.pone.0337913

**Published:** 2026-01-05

**Authors:** Nutcha Pinjaroen, Wirichada Pan-Ngum, Kittiyod Poovorawan, David Wastlund, Fabian Mueller, Peng Lu, Rebecca Sim Shu Yu, Pisit Tangkijvanich

**Affiliations:** 1 Department of Radiology, Faculty of Medicine, Chulalongkorn University, Bangkok, Thailand; 2 Center of Excellence in Hepatitis and Liver Cancer, Faculty of Medicine, Chulalongkorn University, Bangkok, Thailand; 3 Department of Tropical Hygiene, Faculty of Tropical Medicine, Mahidol University, Bangkok, Thailand; 4 Mahidol-Oxford Research Unit, Faculty of Tropical Medicine, Mahidol University, Bangkok, Thailand; 5 Department of Clinical Tropical Medicine, Faculty of Tropical Medicine, Mahidol University, Bangkok, Thailand; 6 Vista Health Ltd, Singapore,; 7 Roche Diagnostics Asia Pacific Ltd., Singapore, Singapore; Helwan University, EGYPT

## Abstract

**Objective:**

Hepatocellular carcinoma (HCC) is the leading cause of cancer-related death in Thailand. However, most Thai patients at high risk of HCC lack access to routine surveillance programs. This study used ultrasound- or biomarker-based screening approaches to assess the cost-utility analysis of routine HCC surveillance in patients with compensated liver cirrhosis (CLC).

**Method:**

The model utilized a Markov-style microsimulation framework to simulate outcomes from alternative HCC surveillance methods for Thai patients. The model was designed to represent Thai patients and healthcare as accurately as possible, and novel Thai patient data was used to estimate treatment and survival associated with screening. Outcomes included diagnostic performance, total costs, and overall health expressed as quality-adjusted life years (QALYs). The incremental cost-effectiveness ratio (ICER) was assessed according to the Thai willingness-to-pay threshold (฿160,000 = 4,800 USD).

**Results:**

Results suggest that routine HCC surveillance is likely cost-effective in Thai patients with CLC. Among the biomarker-based approaches, GAAD score, which combined gender, age, alpha-fetoprotein (AFP), and des-gamma carboxyprothrombin (DCP), was the most cost-effective due to its high detection of HCC while resulting in comparably few false positive diagnoses. Compared to no routine surveillance, GAAD surveillance is suggested to be cost-effective (ICER: $4,631). Compared to ultrasound plus AFP – the recommended standard of care – GAAD is suggested to be dominant, resulting in better overall health at a lower cost.

**Conclusion:**

Bi-annual routine HCC surveillance is suggested to be cost-effective for the Thai healthcare system when used for patients with CLC. Among biomarker-based approaches, GAAD appears to be the most cost-effective and could maximize the benefits of HCC surveillance in high-risk patients.

## Introduction

Primary liver cancer, including hepatocellular carcinoma (HCC) and intrahepatic cholangiocarcinoma (CCA), represents the most common type of cancer in Thailand, accounting for 15.2% of all cancer cases [1]. HCC is one of the most aggressive tumors worldwide, with most cases developing in the background of liver cirrhosis caused by various chronic liver diseases [2]. In Thailand, the main etiological factors of HCC are chronic hepatitis B virus (HBV), followed by chronic hepatitis C virus (HCV) infection and steatotic liver diseases [3–5]. There is a strong association between early HCC detection and the likelihood of effective therapeutic intervention. Several treatment options are available to patients identified with early-stage HCC, and the expected survival typically exceeds 5 years. By contrast, patients detected at advanced stages face limited treatment options and much lower expected survival [6].

As early-stage HCC is asymptomatic, prospective surveillance of high-risk patients is imperative for early-stage HCC detection. Current professional recommendations for HCC surveillance, including the Thailand HCC guideline, suggest screening using ultrasound (US) coupled with serum alpha-fetoprotein (AFP) every 6 months in patients with cirrhosis [2,7]. However, there are limitations to this current standard of care. Meta-analyses have shown that the sensitivity of US plus AFP (US + AFP) for early-stage HCC in cirrhotic patients is relatively low, with a sensitivity of 63% [8]. Additionally, the diagnostic accuracy of US + AFP depends on the skill of the personnel conducting the test, and detection is particularly challenging for obese patients and small lesions (<2 cm) [9]. Moreover, approximately 25% of patients may experience screening-related harm due to false positive results from US reports [10]. Though regular surveillance has been shown to reduce mortality, screening uptake is still relatively low, particularly for patients in rural and low-resource areas where access to ultrasound is limited [11,12]. Given these barriers, many patients are diagnosed only at advanced stages when palliative care is the only available option.

Emerging evidence has indicated that blood-based biomarkers with sufficient diagnostic performance for early-stage cancer have the potential to overcome these barriers and increase early detection of HCC amongst high-risk patients [13]. The commonly validated biomarkers are des-gamma carboxyprothrombin (DCP), also known as protein induced by vitamin K absence-II (PIVKA-II), and *Lens culinaris agglutinin*-reactive AFP (AFP-L3) [13]. Given the heterogeneity of HCC, combined biomarkers incorporating demographic risk factors, including gender and age of the patient, have also been investigated. Among algorithms to date, GALAD (gender, age, AFP-L3, AFP, DCP) score and GAAD (gender, age, AFP, DCP) score have been developed to predict the probability of having HCC in high-risk populations [14,15]. Further, these algorithms have improved early-stage HCC detection compared to AFP for high-risk patients undergoing HCC surveillance [15,16]. Recent data have demonstrated that GALAD and GAAD scores display comparable diagnostic performance in detecting small HCC regardless of etiological factors [15,17]. As such, routine HCC surveillance using these tests may translate into increased early detection, supporting patient referral to potentially curative treatments.

Currently, limited studies have evaluated the cost-effectiveness of these blood-based biomarkers for HCC surveillance as a standard of care. Thus, this analysis aimed to evaluate the cost-effectiveness of routine HCC surveillance using GALAD, GAAD, and PIVKA-II plus AFP (PIVKA-II + AFP) compared to US + AFP or no routine surveillance amongst Thai patients with compensated liver cirrhosis (CLC).

## Methods

### Population and intervention

The analysis targeted screening Thai patients with compensated liver cirrhosis (CLC) aged 40–60 years, in line with the guideline of the Thai Association for the Study of the Liver (THASL), electronically distributed to Thai physicians at https://thasl.org/thasl-guideline/.

### Surveillance options

The analysis compared alternative methods for routine HCC surveillance using bi-annual screening (i.e., every 6 months). US-based methods included US alone and US + AFP, which aligns with current Thai clinical guidelines. Biomarker-based methods included GAAD, GALAD, and PIVKA-II + AFP. To reflect current clinical practice, all methods were compared to both US + AFP and no routine HCC surveillance (‘No surveillance’). For No surveillance, HCC cases would only be symptomatically detected.

### Model description

**Fig 1:**
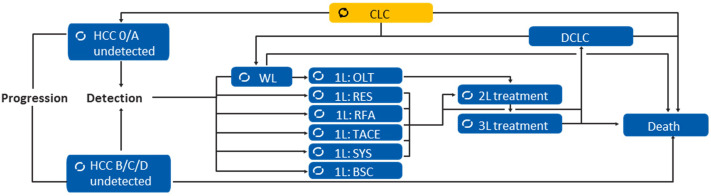
Schematic overview of model health states for the disease progression of patients with CLC.

An economic model was developed in Microsoft Excel to assess the cost-effectiveness of routine bi-annual HCC surveillance using different screening methods in Thai patients. A Markov-style microsimulation model was used to simulate the health, diagnostic outcomes, and treatment of individual patients. The core features of the model have been previously published [18]. The model included health states based on the patient’s liver status, including CLC and decompensated liver cirrhosis (DCLC), HCC status based on the Barcelona-Clinic Liver Cancer (BCLC) staging system (early stages 0/A and late stages B/C/D), and treatments [6]. Simulations were performed using model cycles so that individuals could transition between health states based on conditional probabilities during each cycle and each model cycle represented one surveillance interval of 6 month. An overview of the health states and possible transitions within the model is presented in Fig 1.

For each model cycle, all individuals without known HCC or DCLC were surveyed for HCC using the respective methods. Where HCC was suspected (whether correctly or due to a false positive diagnosis), individuals were referred to confirmatory screening, which consisted of magnetic resonance imaging (MRI) and an accompanying medical visit. Individuals with confirmed HCC diagnosis were referred to treatment, and those without HCC would re-enroll in routine bi-annual surveillance. The diagnostic accuracy of screening differed in the HCC stage. For simplicity, it was assumed that the confirmatory scan had perfect accuracy. On top of detection through routine surveillance, HCC-positive individuals also had a chance of being symptomatically detected to reflect real-world clinical practice. Each individual’s health journey was simulated for each intervention to compare outcomes between methods.

For individuals with confirmed HCC, the model included six treatment options: orthotopic liver transplant (OLT), surgical resection (SR), radiofrequency ablation (RFA), trans-arterial chemoembolization (TACE), systemic treatment (SYS), and best supportive care (BSC). The probability of receiving each treatment depended on the stage of HCC at detection: early vs. late. HCC stages were categorized by the BCLC staging system, where stages 0/A were considered early, and B/C/D were considered late [3,4,6].

Mortality in the model was state-dependent. The individual could progress to death from any state, but the probability increased with deteriorating liver status. Each HCC treatment had its associated survival curve, and the probability of receiving each treatment depended on the HCC stage at detection. Once patients were assigned to a treatment, however, the survival of the individual depended only on the treatment-specific survival.

### Model input data

Inputs for the economic model were obtained through literature reviews and interviews with Thai clinical experts. Preference was given firstly to Thai data and secondly to data from neighboring countries. For diagnostic accuracy, only data that distinguished early- from late-/all-stage HCC was considered. Where available, data from the meta-analysis were preferred over other sources. Where multiple alternative data sources existed, Thai clinical experts were asked to assess the suitability of each input for the Thai clinical context. Key model input data is presented in Table 1, and comprehensive model input data is presented in S1 Appendix.

**Table 1:** Key model inputs for clinical effectiveness, safety, health-related utility, and costs.

Category	Variable name	Sensitivity, Early-stage	Specificity, Early-stage	Sensitivity, All-stage		Specificity, All-stage	Sources
CLC: US	(%) Diagnostic performance, US	45.0%	92.0%	78.0%		92.0%	Tzartzeva et al. (2018) [8]^a^
CLC: US + AFP	(%) Diagnostic performance, US + AFP	63.0%	84.0%	97.0%		84.0%	Tzartzeva et al. (2018) [8]^a^
CLC: US + AFP	(%) Diagnostic performance, US + AFP, used in the scenario analysis	64.0%	84.0%	81.0%		84.0%	Decharatanachart et al (2024) [23]
CLC: GAAD	(%) Diagnostic performance, GAAD	67.4%	86.6%	81.9%		86.6%	Garay et al. (2024) [18]
CLC: GALAD	(%) Diagnostic performance, GALAD	82.1% ‡	81.6% ‡	81.4% ‡		89.1% ‡	Berhane et al (2016) [16]^b^
CLC: PIVKA+AFP	(%) Diagnostic performance, PIVKA + AFP	75.6%	75.9%	86.8%		75.9%	Roche diagnostics, data on file
**HCC treatment distribution for early-stage HCC**							
**Treatment**	**Chulalongkorn University (2024)**	**Sethasine et al. (2023)** [20]^e^			**Somboon et al. (2014)** [24]^ce^		
OLT	0.7%	0%			0.0%		
Resection	22.5%	30.2%			14.4%		
RFA	34.2%	36.1%			21.6%		
TACE	42.1%	33.6%			41.6%		
Systemic Treatment	0.0%	0%			0.00%		
BSC	0.5%	0%			22.4%		
**HCC treatment distribution for late-stage HCC**							
**Treatment**	**Chulalongkorn University (2024)**	**Kitiyakara et al. (2022)** [19]^e^			**Somboon et al. (2014)** [24] ^ce^		
OLT	0.6%	0.0%			0.0%		
Resection	5.5%	4.7%			14.4%		
RFA	2.9%	10.1%			21.6%		
TACE	69.1%	21.1%			41.6%		
Systemic Treatment	0.6%	32.0%			0.00%		
BSC	21.2%	32.0%			22.4%		
**Survival per HCC-stage**							
**Treatment**	**Median survival (months),** **Chulalongkorn University (2024)**				**Median survival (months),** **Reig et al. (2022)** [6]^**e**^		**Median survival (months),** **Sethasine et al. (2023)** [20]^**e**^
OLT	88				60 + ^d^		Not reported
Resection	71.3				60 + ^d^		38.1
RFA	63.8				60 + ^d^		55
TACE	29.2				30		26
Systemic Treatment	6.0				Not reported		Not reported
BSC	3.6				3		Not reported
**Surveillance cost per event ($)**							
**Surveillance**	**Value ($)**				**Source**		
No surveillance	$0				N/A		
US	$24				Chulalongkorn University		
US + AFP	$32				Chulalongkorn University		
GAAD	$35				Chulalongkorn University		
GALAD	$65				Chulalongkorn University		
PIVKA + AFP	$26				Chulalongkorn University		
**Utility**							
**Status**	**Value**				**Source**		
CLC	0.75				Zhang et al (2021) [21]		
DCLC	0.68				Zhang et al (2021) [21]		
HCC undetected	0.64				Zhang et al (2021) [21]		
OLT & Post	0.64				Assumed the same as HCC		
Resection and Post	0.64				Assumed the same as HCC		
RFA & Post	0.64				Assumed the same as HCC		
TACE & Post	0.64				Assumed the same as HCC		
BSC & Post	0.40				Lima et al. (2019) [25]		
Systemic treatment	0.40				Lima et al. (2019)		
Palliative	0.40				Lima et al. (2019)		
**Other**							
Annual DCLC incidence	11.8%	Fleming et al. (2010) [26]					
Annual HCC incidence, CLC	1.95%	Chitapanarux et al. (2015) [3]					
Cost of confirmatory MRI	$359	Riewpaiboon et al. (2014) [27], adjusted for inflation					

^a^Based upon data reported for CLC-patients only from Tzartzeva et al.

^b^Based upon model for Japanese population. The population mixed cirrhotic patients with those with chronic hepatitis B but no cirrhosis; approximately 53.5 of patients in cohort were cirrhotic.

^c^Not reported by HCC-stage at detection.

^d^Reported to be more than 5 years for patients in HCC stages 0/A, with no distinction by treatment type.

^e^Scenario analysis only.

Abbreviations: BSC: best supportive care; CLC: compensated liver cirrhosis; DCLC: decompensated liver cirrhosis; HCC: hepatocellular Carcinoma; OLT: orthotopic liver transplantation; MRI: magnetic resonance imaging; RES: resection; RFA: radiofrequency ablation; SYS: systemic therapy; TACE: transcatheter arterial chemoembolization; WL: waiting list.

### Treatment and survival

To accurately reflect the treatments and expected survival for Thai patients, novel data collected from electronic medical records of Chulalongkorn University was analyzed and implemented into the model. This data includes patients enrolled between 2011 and 2015, with follow-up extending until 2024.

The data included treatments received and survival per BCLC stage 0/A/B/C/D. Treatment type was calculated for early-stage (0/A) and late-stage HCC (B/C/D). Median survival was calculated per treatment type and BCLC stage and then weighted by the proportion of patients within each BCLC stage. To implement survival data into the model, survival curves were fitted for each treatment using an exponential distribution and converted to model cycle intervals. A limitation of the economic model was that the treatment-specific survival curves did not distinguish between early- and late-stage HCC, which was considered acceptable since it may rather overestimate effectiveness at late stage and of no detection rather than of the intervention in scope.

Alternative data sources were used for scenario analyses. Kitiyakara et al. [19] reported real-world data on survival and treatments from Thailand’s universal healthcare coverage (UHC). Sethasine et al. [20] presented survival and treatment data for Thai patients detected with early-stage HCC. Finally, survival curves were fitted for the survival of patients according to BCLC stages [6] used in the current Thailand HCC guideline.

### Quality of life

Utility values were obtained from Zhang et al. [21] in the absence of local utility data for Thai CLC and HCC patients. Since no treatment-specific utility values were reported, the utility for OLT, SR, RFA, and TACE were assumed to be the same as for general HCC. Utility values for systemic treatment and BSC were assumed to be the same as those for palliative care. In the absence of specific utility values, this approach was deemed conservative enough since it would likely underestimate the results of the intervention compared to utility values reported elsewhere. All utility values are presented in S1 Appendix.

### Cost and resource use

All costs were reported in American dollars ($) (1.00 ฿ = 0.03 USD). screening costs for US, AFP, PIVKA-II, GAAD, and GALAD were obtained from the price list of services offered by the Faculty of Medicine, Chulalongkorn University; a scenario using price parity between GAAD and GALAD is presented in S4 Appendix. The annual cost of CLC, DCLC, and true positive for HCC (confirmatory were obtained from the study by Sangmala et al. [4].

The cost of SR, RFA, TACE, and BSC were obtained from DRG Chulabhorn Hospital, as reported in the literature by Chanree et al. [22] and confirmed by the Chulalongkorn team. The authors also reported the drug costs for systemic treatment. For this analysis, it was assumed that the cost of systemic treatment would include both overall costs for BSC and the drug cost. The cost of OLT per operation and the cost of post-OLT follow-up (year 1) were obtained from Sangmala et al. [4]. This data was validated against data from Chulalongkorn University to ensure that it would accurately reflect contemporary Thai clinical practice.

### Analytical setting

Model outcomes included diagnostic outcomes, total costs, and quality-adjusted life years (QALYs). Costs and QALYs were measured over a lifetime horizon, and incremental cost-effectiveness per QALY was calculated. The discount rate for both costs and health outcomes was 3%, and a cost-effectiveness threshold of $4,800/ QALY (฿160,000/ QALY) gained was used, according to Thai Health Technology Assessment (HTA) guidelines. A healthcare sector cost perspective was applied.

Base case results were based upon 50,000 microsimulations to limit the influence of randomness in patient draw. Scenario, one-way, and probabilistic sensitivity analyses (PSA) were performed to evaluate the uncertainty and variation of the model’s results. The one-way sensitivity analysis varied input parameters by ±20%. For the PSA, parametric uncertainty was captured using normal and beta distributions for clinical and utility parameters and gamma distribution for cost parameters. Cost-effectiveness acceptability curves (CEAC) were used to analyze the probability of cost-effective interventions at various thresholds. Scenario and sensitivity analyses were based on 10,000 microsimulations to maintain computational efficiency.

## Results

### Treatment & survival

The team at Chulalongkorn University contributed relevant data to determine model inputs for treatment allocation and associated survival rates. This analysis based on data from 1,132 Thai HCC patients is used to determine treatment type and survival analyses. These included 78.9% male and 21.1% female patients. Patients were 15–96 years old, with a median age of 60.3 (SD: 11.8 years). Treatment type and survival by early-stage HCC (BCLC: 0/A) and late-stage HCC (BCLC: B/C/D) are presented in Table 2. Patients detected during early-stage HCC were most treated with SR (22.1%), RFA (33.6%), or TACE (41.4%). By contrast, patients detected during late-stage HCC were mainly treated with only TACE (62.4%) or best supportive care (19.1%). The analysis’s median survival by treatment type aligned well with those reported in the BCLC treatment recommendation [6].

**Table 2:** Treatment type and treatment-specific survival per HCC stage.

Treatment type		
	Early-stage HCC (n = 411)	Late-stage HCC (n = 721)
OLT	3 (0.73%)	4 (0.55%)
Resection	91 (22.14%)	36 (4.99%)
RFA	138 (33.58%)	19 (2.64%)
TACE	170 (41.36%)	450 (62.41%)
Systemic therapy	0 (0.0%)	4 (0.55%)
Best supportive care	2 (0.49%)	138 (19.14%)
Other ^a^	7 (1.70%)	70 (9.71%)
**Median survival**		
	**Chulalongkorn data** **(months)**	**Current THASL guidelines** **(months)**
OLT	88.0	60 ^b^
Resection	71.8	60 ^b^
RFA	63.8	60 ^b^
TACE	29.2	30
Systemic therapy	6	N/A
Best supportive care	3.6	3

^a^Other treatment types included PEIT, Bland, Y-90 and Radiation.

^b^Data did not distinguish survival between OLT, resection and RFA.

Abbreviations: HCC: hepatocellular Carcinoma; OLT: orthotopic liver transplantation; RFA: radiofrequency ablation; TACE: transcatheter arterial chemoembolization.

### Cost & health outcomes

Results show that all methods for routine surveillance would increase the detection of HCC. Per 10,000 screened individuals with CLC, an estimated 771–1,003 cases could be detected during early-stage HCC over the routine screening period (years 40–60). This meant that 71–93% of all HCC cases were detected during early-stage HCC, depending on the surveillance method. Biomarker-based surveillance was more effective than US-based surveillance for detecting HCC, with GALAD identifying most cases. A downside of routine HCC surveillance is the risk of increased false positive diagnoses. US (alone) resulted in the fewest false positive diagnoses, followed by GAAD. By contrast, PIVKA + AFP and GALAD yielded the highest false positive diagnoses. Results are shown in Table 3.

**Table 3:** Costs, QALYs, and diagnostic outcomes, all interventions, base case, CLC patients.

	No surveillance	US	US + AFP	GAAD	GALAD	PIVKA+AFP
Early-stage detection	0 (0%)	771 (71%)	913(84%)	942 (87%)	1,003 (93%)	982 (91%)
Late-stage detection	0 (0%)	158 (15%)	88 (8%)	64 (6%)	28 (3%)	44 (4%)
Detected symptomatically	1,083 (100%)	154 (14%)	82 (8%)	77 (7%)	51 (5%)	57 (5%)
TP	0	929	1,001	1,006	1,032	1,026
TN	0	98,289	89,726	92,502	87,175	81,099
FN	0	974	524	436	218	295
FP	0	8,476	17,039	14,264	19,590	25,666
Total cost ($) ^a^	$903	$1,589	$1,952	$1,894	$2,352	$2,161
Total LYs †	8.89	9.19	9.22	9.23	9.24	9.23
Total QALY †	6.38	6.56	6.59	6.59	6.60	6.59
Total cost ($, undiscounted)	$1,209	$1,997	$2,412	$2,345	$2,866	$2,648
Total LYs (undiscounted)	11.37	11.80	11.85	11.85	11.87	11.87
Total QALY (undiscounted)	8.11	8.38	8.41	8.42	8.43	8.42
ICER vs ‘No surveillance’	Na	$3,629	$4,957	$4,631	$6,558	$5,752
ICER vs US + AFP ($ per QALY)	loss of QALYs $4,957	loss of QALYs $15,990	Na	Dominant -$26,049	$42,887	$29,828

^a^Results discounted using a 3.0% discount rate.

Abbreviations: AFP: Alpha-fetoprotein; FN: False negative; FP: False positive; GAAD: Elecsys® GAAD; PIVKA: antagonist; GALAD: Glycine, Alpha-Fetoprotein, and Des-Gamma-Carboxy Prothrombin-Liver Cancer Early Detection; TN: True negative; TP: True positive; US: ultrasound.

‘No surveillance’ was estimated to be the least costly strategy, with a total cost of $903 per screened individual. This was due to the absence of primary surveillance costs, the low cost of confirmatory tests due to this method yielding no false positive diagnoses, and low and short duration of associate best supportive care. This was followed by US (alone) and GAAD. Due to its higher false positive rate, as a consequence of its relatively lower suggested specificity, GALAD was more costly than GAAD, even at price parity (S4 Appendix). For all surveillance methods, the most considerable costs were (in descending order): treatment of DCLC, cost of false positive diagnoses (i.e., unnecessary confirmatory testing with MRI), cost of surveillance itself, and cost of HCC treatment. For no routine surveillance, the main costs were for the treatment of DCLC and the palliative treatment of HCC patients.

**Fig 2:**
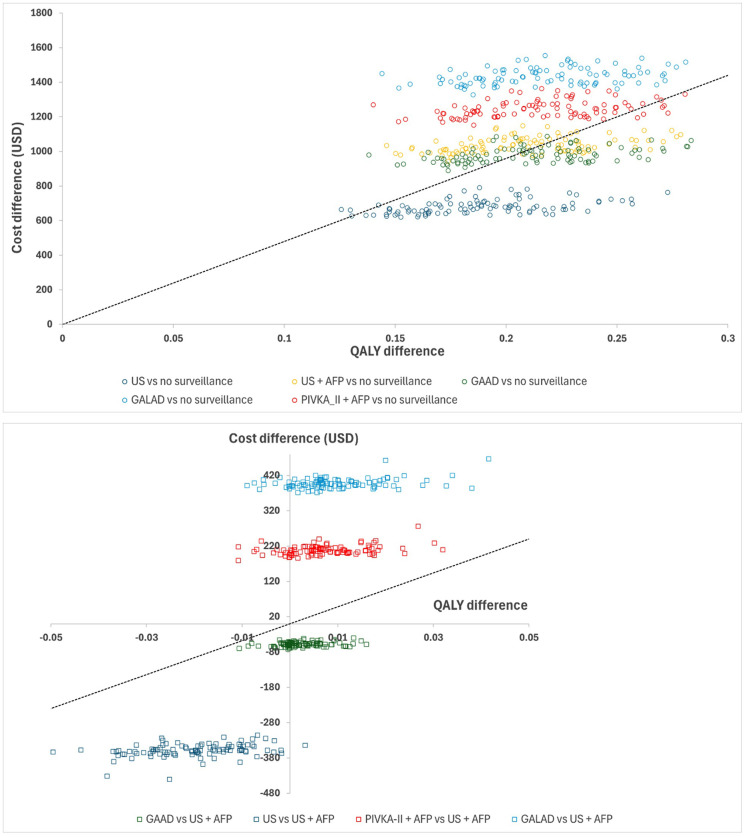
Cost-effectiveness plane, all major surveillance strategies vs. no surveillance (top) and US + AFP (bottom).

For health outcomes, all surveillance methods improved QALYs substantially compared to no surveillance. In terms of cost-effectiveness, US (alone) and GAAD had the lowest ICERs per QALY vs no surveillance: $3,629 and $4,631, respectively. Compared to US + AFP as the standard of care, GAAD screening was dominant with a decreased cost of $59 and an additional 0.002 QALYs per person. The cost-effectiveness plane for all major surveillance strategies vs ‘No surveillance’ is presented in Fig 2. The CEAC for GAAD vs ‘No Surveillance’ is presented in S2 Appendix; it was estimated that GAAD had a higher than 57% probability of being cost-effective at a threshold of $4,800.

### Sensitivity, scenario, and budget impact analyses

The cost-effectiveness of routine HCC surveillance was assessed for various scenarios, including alternative screening age and survival data. Generally, the difference between routine surveillance methods was small between scenarios, with GAAD remaining either dominant or cost-effective compared to US + AFP. Compared to ‘No surveillance’, routine surveillance benefited from scenarios with higher survival or where more patients received effective treatments. The probabilistic sensitivity analysis results supported that HCC surveillance using GAAD in CLC patients is cost-effective compared to no routine surveillance and dominant compared to US + AFP.

A budget impact analysis (BIA) was conducted to explore the expected impact of routine surveillance with GAAD compared to US + AFP. Results from the budget impact analysis are presented in S3 Appendix. Overall, estimations suggest switching to GAAD instead of US + AFP would have low impact on the overall healthcare costs, with adoption of GAAD among newly diagnosed Thai CLC patients being slightly cost-saving during the first 5 years. The main cost-saving would mainly stem from avoiding false positive diagnoses and their associated costs; by contrast, both HCC surveillance and treatment costs are expected to increase.

## Discussion

This analysis has compared the value of routine HCC surveillance using biomarker-based screening to the current standard of care in Thailand from a cost-effectiveness perspective. Surveillance improves early-stage HCC detection, which enables more effective care and improved survival. The analysis suggests that routine bi-annual HCC surveillance could be cost-effective, but other methods may be preferred over ‘US + AFP’. From a cost-effectiveness perspective, GAAD was the preferred method of the biomarker-based approaches. Using US only would be cost-effective due to its low false positive detection rate, but it would likely fail to detect many prospective HCC cases in addition to the general/qualitative limitation described above.

Compared to no routine surveillance, routine screening using GAAD was associated with an additional 1,083 detected HCC cases, with 87% of these being detected during early-stage HCC. Subject to effective treatment, this surveillance strategy was associated with an increase of 0.214 QALYs per screened individual and an ICER per QALY of $4,631 for the CLC population. When compared to routine surveillance using US + AFP – the suggested standard of care in Thailand – GAAD was estimated to be a dominant strategy, i.e., associated with lower total cost (-$58.57) and better overall health outcomes (+0.002) per patient. Similar outcomes were also observed in the UK, where GAAD was cost-effective compared with US alone or US + AFP [18]. Screening with GALAD was estimated to increase overall QALYs slightly, but the additional costs do not appear to be economically justified within the current Thai acceptability criteria compared to other surveillance strategies, mainly due to its increased false positive detection rate and its associated costs. Further, most of the HCC cases missed by GAAD were detected during the subsequent screening round instead.

While all methods of routine HCC surveillance were found to improve HCC detection substantially, their ability to avoid false positive diagnoses differed significantly. For patients receiving an initial positive screening test, follow-up testing for confirmatory diagnosis is necessary. Such follow-up testing is costly, requiring further medical visits and often costly screening methods, e.g., MRI or computed tomography (CT) scan. Though GALAD identified the highest number of HCC cases, GAAD – with its relatively high specificity during early-stage HCC – suggests a better balance between a high detection rate and a comparatively low false positive rate. Given the high costs of confirmatory testing, GAAD was preferred from a cost-effectiveness perspective.

It is important to note that the value of routine HCC surveillance increases when effective treatments (OLT, SR, and RFA) are given to patients detected with early-stage HCC. Observational evidence from Thailand has previously shown limited survival of HCC patients, especially in rural areas, which would imply a limited value of diagnosis [19]. However, in the absence of routine HCC surveillance, most patients in this data were detected with late-stage HCC. By contrast, this study was able to distinguish treatment type and survival by early- and late-stage HCC, which shows that earlier diagnosis is linked to substantial increases in survival. The novel data from Thai patients used for this study aligned well with the expected survival reported in the Thailand HCC guideline, e.g., 63.8 vs. 60 months for RFA, 29.2 vs. 30 months for TACE, and 3.6 vs. 3 months for BSC [5]. The median survival for OLT and resection was even better: 88.0 and 71.8 months, respectively. This shows that the Thai healthcare system may offer great survival benefits for patients whose HCC may be detected during the early stages.

Although having a higher ICER vs no surveillance than US alone, surveillance using GAAD may still be a highly considerable surveillance strategy for Thailand-wide patient access. The US alone yielded worse overall health outcomes than the currently recommended standard of care (US + AFP). Further, compared to US-based methods, biomarker-based HCC surveillance could more easily be upscaled to all Thai regions since the diagnostic accuracy is a ‘One Stop Service’ for testing that is not dependent on performance skills and may be less dependent on US capacity constraints and availability.

The value of routine HCC surveillance using biomarker-based methods will likely differ across Thai regions. In urban settings, their implementation is likely more accessible, and effective treatment is already available for additionally detected early-stage HCC patients. However, GAAD may offer a greater advantage in rural settings over US-based screening due to the limited availability of skilled ultrasonographers and equipment. The ‘Cancer Everywhere policy’ in Thailand allows the referral of patients in rural areas to tertiary referral hospitals. Access to regular screening tests can bring higher benefits and efficiency to rural areas, following the ‘Cancer Warrior initiative in Liver Cancer’ to build up community care centers. While this analysis relied on the meta-analysis’s diagnostic accuracy data for US and US + AFP [8], real-world accuracy would likely be worse if extended to providers with less screening competence.

The analysis utilised real-world incidence rates for HCC from Chitapanarux et al. [3], consistent with other data sources [28]. However, recent studies have shown that antiviral treatment, such as tenofovir or entecavir for chronic hepatitis B, and direct-acting antivirals for chronic hepatitis C may reduce the risk of HCC development [29,30], the major aetiologies for CLC in Thailand [5]. Therefore, the incidence rate of HCC may decline in the future. Whilst this would not affect the relative value of different methods for HCC surveillance, it is possible that the overall costs associated with HCC could be lower in the future than what this study has estimated.

One limitation of this study was that diagnostic accuracy data for the relevant comparators were obtained from multiple sources. Though accurate data were obtained from meta-analyses, biases related to small differences in settings and study designs cannot be ruled out. Unfortunately, no head-to-head comparison of the accuracy of all relevant comparators (distinguishing between early-stage and all-stage HCC) exists at present. For US-based methods, the extent to which the accuracy may deteriorate if extended across more providers is also uncertain. A second limitation was that differences in survival between early-stage and late-stage diagnosis were only indirectly captured through the share of patients receiving each treatment; once assigned to a treatment, the survival was the same whether the initial diagnosis was made in early-stage or late-stage HCC. Third, non-financial costs from false positive diagnoses (e.g., patient worry) were unaccounted for, so the value of avoiding false positive diagnoses may be even more significant in clinical practice. Fourth, the Thai healthcare system is heterogeneous, and the prices for HCC surveillance may differ across its regions and provinces. Without credible surveillance costs from all relevant healthcare settings, particularly in rural areas, the cost data obtained for this analysis were considered a conservative and suitable assumption. Finally, this study estimated higher survival among patients receiving no routine surveillance than previous comparable studies in Thailand. All of the limitations above were partly accounted for through the probabilistic sensitivity analysis as well as through making conservative assumptions in areas of uncertainty which would rather underestimate the value of surveillance.

Previous research has suggested that routine bi-annual HCC surveillance should be offered to Thai patients with CLC, aged 40–60 years. The findings from this study support this concept and suggest that biomarker-based methods for routine HCC surveillance – particularly GAAD – could improve health outcomes at feasible costs, particularly in healthcare settings where access to US remains limited.

## Supporting information

S1 Appendix Model input data. (DOCX)

S2 Appendix Extended results from cost-effectiveness analysis. (DOCX)

S3 Appendix Budget impact analysis. (DOCX)

S4 Appendix Comparison of GAAD vs GALAD. (DOCX)
