# Supplementary material for: Economic evaluation of biomarker-based surveillance for Hepatocellular Carcinoma in Thai patients with Compensated Liver Cirrhosis
Source: PLoS One. 2026 Jan 5;21(1):e0337913. doi: 10.1371/journal.pone.0337913 (PMC12768342; doi:10.1371/journal.pone.0337913)
Supplement: S1 Appendix — (DOCX) [file pone.0337913.s001.docx]

# Appendix 1: Model input data

**S1 Table. Cost data inputs used in the analysis**

| **Category** | **Variable name** | **Base  value ($)** | **Inferior  value ($)** | **Superior  value ($)** | **Sources** |
| --- | --- | --- | --- | --- | --- |
| **Surveillance costs** | US | $24 | $19.2 | $28.8 | Thai reimbursement price list |
|  | US+AFP | $32 | $25.6 | $38.4 | Thai reimbursement price list |
|  | GAAD | $35 | $28 | $42 | Estimated reimbursement price (informed through request with Roche) |
|  | GAAD+US | $59 | $47.2 | $70.8 | Estimated reimbursement price (informed through request with Roche) |
|  | Elecsys PIVKA II | $20 | $16 | $24 | Estimated reimbursement price (informed through request with Roche) |
|  | Elecsys PIVKA II + Elecsys AFP | $26 | $20.8 | $31.2 | Estimated reimbursement price (informed through request with Roche) |
|  | Elecsys AFP | $6 | $4.8 | $7.2 | Estimated reimbursement price (informed through request with Roche) |
|  | GALAD  *Price parity with GAAD is explored in the scenario analysis | $68 | $54.4 | $81.6 | Estimated cost based on input from Chulalongkorn University |
|  | True positive for HCC (confirmatory) | $345 | $276 | $414 | Sangmala et al. (2014)^[1]^, assuming 1 MRI for confirmation |
|  | False positive for HCC | $345 | $3276 | $414 | Sangmala et al. (2014)^[1]^, assuming 1 MRI for confirmation |
|  | Incidental diagnosis | $345 | $276 | $414 | Sangmala et al. (2014)^[1]^, assuming 1 MRI for confirmation |
|  | Follow-up after HCC, per cycle | $64 | $51.2 | $76.8 | Riewpaiboon et al. (2014)^[2]^ |
| **Treatment costs** | OLT, per operation | $15,417 | $12,334 | $18,500 | DRG Chulabhorn Hospital, 2013 (from Sangmala et al (2014)^[1]^)^a^; verified with Chulalongkorn University |
|  | Post-OLT follow-up (year 1) | $2,562 | $2,050 | $3,074 | Siriraj from Sangmala et al (2014)^[1)^, (DMSIC, 2013)^a^; verified with Chulalongkorn University |
|  | Post-OLT follow-up (year 2+) | $ 3,496 | $2,797 | $4,195 | [Thongsawat et al (2014)](https://pubmed.ncbi.nlm.nih.gov/29702917/)^[3]^ ; verified with Chulalongkorn University |
|  | Resection | $1,867 | $1,494 | $2,240 | Chanree et al. (2022)^[4]^ DRG Chulabhorn Hospital; verified with Chulalongkorn University |
|  | RFA | $2,422 | $1,938 | $2,906 | Chanree et al. (2022)^[4]^ DRG Chulabhorn Hospital; verified with Chulalongkorn University |
|  | TACE | $1,880 | $1,504 | $2,256 | Chanree et al. (2022)^[4]^ DRG Chulabhorn Hospital; verified with Chulalongkorn University |
|  | BSC, per month | $80 | $64 | $96 | Chanree et al. (2022)^[4]^ DRG Chulabhorn Hospital; verified with Chulalongkorn University |
|  | Systemic treatment, annual | $1,010 | $808 | $1,212 | Chanree et al. (2022)^[4]^ DRG Chulabhorn Hospital; verified with Chulalongkorn University |

^a^Adjusted for inflation: CPI2013=109.3; CPI2021=113.7

**S2 Table. Summary of the clinical inputs used in the analysis, base case**

| **Category** | **Variable name** | **Base  value** | **Inferior  value** | **Superior  value** | **Sources** |
| --- | --- | --- | --- | --- | --- |
| **Start of surveillance** | (Y) Age, at start of surveillance, CLC | 40 | 30 | 50 | Thailand HCC guideline (2021)^[5]^, Clinical experts |
| **Upper Age limit** | (Y) Age, upper limit for the surveillance. | 60 | 50 | 70 | Thailand HCC guideline (2021) ^[5]^ |
| **CLC aetiology** | (%) ALD aetiology in CLC^a^ | 15.43% | 15.43% | 15.43% | Poovorawan et al.(2015)^[6]^ |
|  | (%) HBV aetiology in CLC^a^ | 46.61% | 46.61% | 46.61% | Poovorawan et al.(2015) ^[6]^ |
|  | (%) HCV aetiology in CLC^a^ | 30.97% | 30.97% | 30.97% | Poovorawan et al.(2015) ^[6]^ |
|  | (%) NAFLD aetiology in CLC^a^ | 6.99% | 6.99% | 6.99% | Poovorawan et al.(2015) ^[6]^ |
| **Transition probability from CLC to HCC** | (%) HCC, annual incidence in CLC (ALD) | 1.95% | 1.56% | 2.34% | Chitapanarux et al. (2015)^[7]^ |
|  | (%) HCC, annual incidence in CLC (HBV) | 1.95% | 1.56% | 2.34% | Chitapanarux et al. (2015) ^[7]^ |
|  | (%) HCC, annual incidence in CLC (HCV) | 1.95% | 1.56% | 2.34% | Chitapanarux et al. (2015) ^[7]^ |
|  | (%) HCC, annual incidence in CLC (NAFDL) | 1.95% | 1.56% | 2.34% | Chitapanarux et al. (2015) ^[7]^ |
| **Incidence of DCLC** | (%) DCLC, annual incidence in CLC (ALD) | 11.8% | 9.44% | 14.16% | Fleming et al. (2010) ^[8]^ |
|  | (%) DCLC, annual incidence in CLC (HBV) | 11.8% | 9.44% | 14.16% | Fleming et al. (2010) ^[8]^ |
|  | (%) DCLC, annual incidence in CLC (HCV) | 11.8% | 9.44% | 14.16% | Fleming et al. (2010) ^[8]^ |
|  | (%) DCLC, annual incidence in CLC (NAFDL) | 11.8% | 9.44% | 14.16% | Fleming et al. (2010) ^[8]^ |
| **Incidental detection** | (%) Annual incidental detection HCC 0.A | 0.0% | 0.0% | 0.0% | Assumption; no data on incidental detection was found for Thailand or nearby markets; thus, it is considered negligible in the base case, assuming equal impact across strategies |
|  | (%) Annual incidental detection HCC BCD | 0.0% | 0.0% | 0.0% | Assumption; no data on incidental detection was found for Thailand or nearby markets; thus, it is considered negligible in the base case, assuming equal impact across strategies |
| **Transition probability from DCLC to HCC** | (%) Patients with DCLC after HCC treatment | 15.5% | 12.4% | 18.6% | [Kondo et al. (2022](https://journals.plos.org/plosone/article?id=10.1371/journal.pone.0261619))^[9]^ |
| **HCC treatments for EARLY detected**  ***base case scenario** | (%) OLT_early^a^ | 0.7% | 0.7% | 0.7% | Chulalongkorn University |
|  | (%) Resection_early^a^ | 22.5% | 22.5% | 22.5% | Chulalongkorn University |
|  | (%) RFA_early^a^ | 34.2% | 34.2% | 34.2% | Chulalongkorn University |
|  | (%) TACE_early^a^ | 42.1% | 42.1% | 42.1% | Chulalongkorn University |
|  | (%) Systemic Treatment_early^a^ | 0.0% | 0.00% | 0.00% | Chulalongkorn University |
|  | (%) BSC_early^a^ | 0.5% | 0.5% | 0.5% | Chulalongkorn University |
| **HCC treatments for LATE detected (urban)** | (%) OLT_late^a^ | 0.6% | 0.6% | 0.6% | Chulalongkorn University |
|  | (%) Resection_late^a^ | 5.5% | 5.5% | 5.5% | Chulalongkorn University |
|  | (%) RFA_late^a^ | 2.9% | 2.9% | 2.9% | Chulalongkorn University |
|  | (%) TACE_late^a^ | 69.1% | 69.1% | 69.1% | Chulalongkorn University |
|  | (%) Systemic Treatment_late^a^ | 0.6% | 0.6% | 0.6% | Chulalongkorn University |
|  | (%) BSC_late^a^ | 21.2% | 21.2% | 21.2% | Chulalongkorn University |
| **HCC treatments for LATE detected (rural)** | (%) OLT_late^a^ | 0.0% | 0.0% | 0.0% | Kitiyakara et al. (2022)^[10]^, nationwide |
|  | (%) Resection_late^a^ | 1.6% | 1.6% | 1.6% | Kitiyakara et al. (2022) et al. (2010) ^[8]^, nationwide |
|  | (%) RFA_late^a^ | 3.2% | 3.2% | 3.2% | Kitiyakara et al. (2022) et al. (2010) ^[8]^, nationwide |
|  | (%) TACE_late^a^ | 6.1% | 6.1% | 6.1% | Kitiyakara et al. (2022) et al. (2010) ^[8]^ , nationwide |
|  | (%) Systemic Treatment_late^a^ | 44.6% | 44.6% | 44.6% | Kitiyakara et al. (2022) et al. (2010) ^[8]^ , nationwide |
|  | (%) BSC_late^a^ | 44.6% | 44.6% | 44.6% | Kitiyakara et al. (2022) et al. (2010) ^[8]^ , nationwide |
| **Waiting list time for OLT** | OLT waiting time (in 6-mo cycles) | 1 | 1 | 1 | Assumption |
| **%DCLC -> OLT** | (%) DCLC, listed for OLT | 5% | 4% | 6% | Assumption |
| **Compliance, US** | (%) Compliance rate, US | 100% | 100% | 100% | Assumption |
| **Compliance, US+AFP** | (%) Compliance rate, US+AFP | 100% | 100% | 100% | Assumption |
| **Compliance, GAAD** | (%) Compliance rate, GAAD | 100% | 100% | 100% | Assumption |
| **Compliance, GAAD+US** | (%) Compliance rate, GAAD+US | 100% | 100% | 100% | Assumption |
| **Compliance, PIVKA+AFP** | (%) Compliance rate, PIVKA II+AFP | 100% | 100% | 100% | Assumption |
| **Utilities** | (QoL) CLC | 0.75 | 0.60 | 0.90 | Zhang et al (2021)^[11]^ |
|  | (QoL) DCLC | 0.68 | 0.55 | 0.82 | Zhang et al (2021) ^[11]^ |
|  | (QoL) HCC undetected | 0.64 | 0.51 | 0.77 | Zhang et al (2021) ^[11]^ |
|  | (QoL) OLT & Post | 0.64 | 0.51 | 0.77 | Assumed the same as HCC |
|  | (QoL) Resection and Post | 0.64 | 0.51 | 0.77 | Assumed the same as HCC |
|  | (QoL) RFA & Post | 0.64 | 0.51 | 0.77 | Assumed the same as HCC |
|  | (QoL) TACE & Post | 0.64 | 0.51 | 0.77 | Assumed the same as HCC |
|  | (QoL) BSC & Post | 0.40 | 0.32 | 0.48 | Lima et al. (2019)^[12]^ |
|  | (QoL) Systemic treatment | 0.40 | 0.32 | 0.48 | Lima et al. (2019) ^[12]^ |
|  | (QoL) Palliative | 0.40 | 0.32 | 0.48 | Lima et al. (2019) ^[12]^ |

Note: In the absence of compliance data specific to each screening type, previous model assumptions (100% compliance, per Sangmala et al. (2014)^[1]^) were applied for all strategies. While no strategy is likely to achieve full compliance, the results may be conservative, assuming greater accessibility for biomarker-based approaches.
^a^Not varied from the base case value in DSA and PSA, since the probability of all options need to sum to 100%.
