# Supplementary material for: Economic evaluation of biomarker-based surveillance for Hepatocellular Carcinoma in Thai patients with Compensated Liver Cirrhosis
Source: PLoS One. 2026 Jan 5;21(1):e0337913. doi: 10.1371/journal.pone.0337913 (PMC12768342; doi:10.1371/journal.pone.0337913)
Supplement: S2 Appendix — (DOCX) [file pone.0337913.s002.docx]

# Appendix 2: Extended results from cost-effectiveness analysis

**S3 Table. Scenario analyses for GAAD vs. US + AFP, showing costs (USD) and health outcomes per screened individual**

|  | **US + AFP** | | **GAAD** | | **GAAD vs. US + AFP** | | |
| --- | --- | --- | --- | --- | --- | --- | --- |
| **Scenario** | **Cost (**$**)** | **QALYs** | **Cost (**$**)** | **QALYs** | **Incremental cost (**$**)** | **Incremental QALYs** | **ICER ($)** |
| Base case | $1,952 | 6.587 | $1,894 | 6.589 | -$58.56 | 0.002 | Dominant, -$26,049 |
| No discounting of QALYs | $2,431 | 8.413 | $2,365 | 8.415 | -$66.01 | 0.002 | Dominant  -$32,034 |
| Screening: 40-50 years only | $1,841 | 6.551 | $1,821 | 6.553 | -$53.52 | 0.001 | Dominant, -$38,891 |
| Screening: 50-60 years only | $1,872 | 6.548 | $1,818 | 6.549 | -$53.52 | 0.001 | Dominant, -$38,891 |
| Real-world compliance rate^a^ | $1,354 | 6.489 | $1,334 | 6.491 | -$19.84 | 0.002 | Dominant, -$11,175 |
| Alternative utility values^b^ | $1,961 | 6.065 | $1,903 | 6.065 | -$58.08 | 0.000 | Dominant, -$3,535,378 |
| Alternative survival data: Reig et al., (2022)^[13]^ | $1,960 | 6.554 | $1,902 | 6.555 | -$58.21 | 0.001 | Dominant, -$58,086 |
| Alternative survival data: Sethasine et al (2023)^[14]^ | $1,955 | 6.494 | $1,897 | 6.495 | -$57.84 | 0.001 | Dominant,  -$52,530 |
| Real-world late-stage outcomes^c^ | $1,956 | 6.642 | $1,900 | 6.646 | -$56.54 | 0.004 | Dominant  -$14,184 |
| Alternative diagnostic performance of US+AFP: Decharatanachart et al., (2024)^[15]^ | $1,960 | 6.568 | $1,903 | 6.570 | -$57.30 | 0.002 | Dominant  -$32,064 |

^a^36.2% for both interventions, based upon Rattanasupar et al. (2021)^[1, 16]^.
^b^Utility values from Sangmala et al (2014)^[1]^: all non-HCC stage: 0.68, all HCC stage: 0.38
^c^Late-stage HCC treatment distribution and survival data for TACE and BSC from Kitiyakara et al., (2022)^[10]^

**S1 Fig. Cost-effectiveness acceptability curve for routine HCC surveillance using GAAD compared to no routine surveillance in patients with CLC**


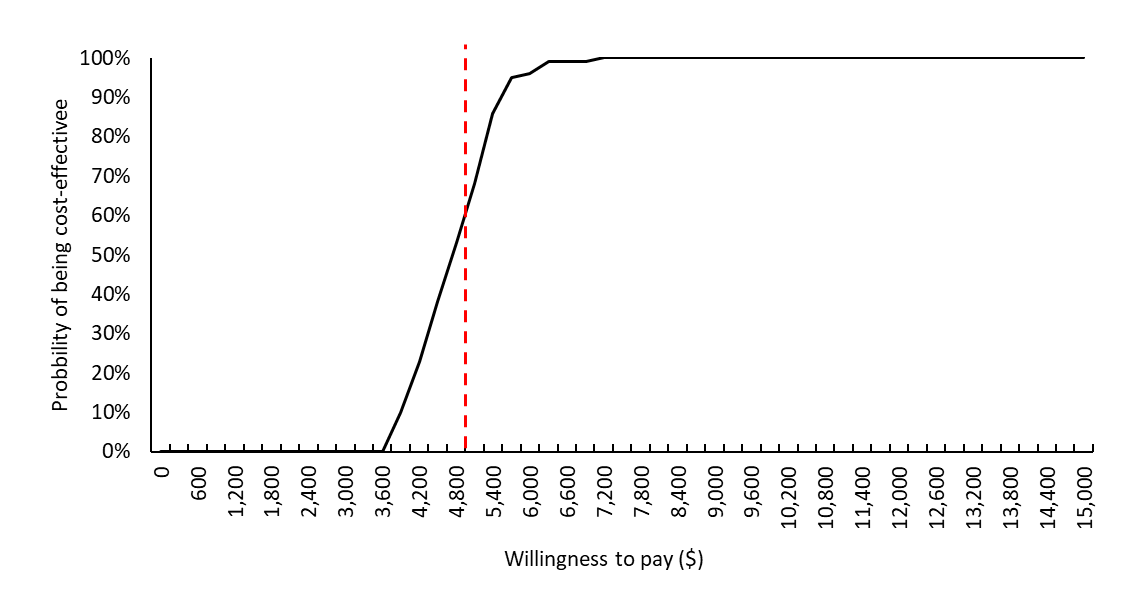


Note: Red line denotes willingness-to-pay threshold of $4,800 per QALY gained
CLC: compensated liver cirrhosis, QALY: quality-adjusted life year
