# Supplementary material for: Economic evaluation of biomarker-based surveillance for Hepatocellular Carcinoma in Thai patients with Compensated Liver Cirrhosis
Source: PLoS One. 2026 Jan 5;21(1):e0337913. doi: 10.1371/journal.pone.0337913 (PMC12768342; doi:10.1371/journal.pone.0337913)
Supplement: S3 Appendix — (DOCX) [file pone.0337913.s003.docx]

# Appendix 3: Budget impact analysis

The cost-effectiveness analysis (CEA) provided has shown that bi-annual HCC surveillance using GAAD is likely to be cost-effective compared to the current standard-of-care (SoC): Ultrasound plus AFP (US + AFP). However, to assess the feasibility of using biomarker-based HCC surveillance in Thailand, the overall budget impact from adoption of GAAD-based surveillance should also be considered. Unlike the CEA, an budget impact analysis (BIA) considers not only the cost per individual screened, but the overall cost associated with GAAD-based surveillance, which depends on the number of individuals targeted. For this BIA, a 5-year time horizon was used, comparing bi-annual surveillance with GAAD to ‘US + AFP’. The applied perspective focuses only on costs.

The total population of Thailand was assumed to be 71,702,435, and the incidence of CLC was 32.07 per 100,000 people^[17, 18]^. Given this, it was estimated that approximately 22,995 new Thai CLC patients would emerge annually. The market share among new patients for GAAD-based surveillance was estimated to be 20% for year 1, 30% (year 2), 40% (year 3), 45% (year 4) and 50% (year 5). For simplicity, all patients not receiving GAAD were assumed to be screened using the current Thai SoC: ‘US + AFP’. It was further assumed that the annual discontinuation rate from the screening program was 5%, to reflect expected drop-out following development of DCLC or HCC, or death. The estimated number of new patients for the intervention is shown in Table S4. For the SoC, all patients were assumed to received screening using ‘US + AFP’

S4 Table. Estimated number of new patients per year eligible for HCC surveillance, intervention

| **GAAD surveillance** | Year 1 | Year 2 | Year 3 | Year 4 | Year 5 |
| --- | --- | --- | --- | --- | --- |
| 1st year patients | 4,599 | 6,899 | 9,198 | 10,348 | 11,498 |
| 2nd year patients | 0 | 4,369 | 6,554 | 8,738 | 9,830 |
| 3rd year patients | 0 | 0 | 4,151 | 6,226 | 8,301 |
| 4th year patients | 0 | 0 | 0 | 3,943 | 5,915 |
| 5th year patients | 0 | 0 | 0 | 0 | 3,746 |
| **‘US + AFP’ surveillance** |  |  |  |  |  |
| 1st year patients | 18,396 | 16,097 | 13,797 | 12,647 | 11,498 |
| 2nd year patients | 0 | 17,476 | 15,292 | 13,107 | 12,015 |
| 3rd year patients | 0 | 0 | 16,602 | 14,527 | 12,452 |
| 4th year patients | 0 | 0 | 0 | 15,772 | 13,801 |
| 5th year patients | 0 | 0 | 0 | 0 | 14,984 |

The total cost per patient for each intervention was estimated using the same cost-effectiveness model as used for the CEA. However, an extension was used which allowed results to be presented by year. Overall cost per patient were based upon 10,000 microsimulations and is presented in Table S5. Note that the random patient sample for the BIA differed from that of the CEA, hence small deviations in overall cost between the two analyses are possible.

S5 Table. Estimated cost by category per surveillance type and year (years 1-5)

| **Cost per patient and year, GAAD** | **Year 1** | **Year 2** | **Year 3** | **Year 4** | **Year 5** | **Total** |
| --- | --- | --- | --- | --- | --- | --- |
| Surveillance | $63.00 | $53.65 | $44.73 | $36.87 | $30.66 | $228.92 |
| HCC treatment | $28.19 | $39.16 | $31.25 | $26.12 | $21.12 | $145.84 |
| False positives | $103.70 | $82.99 | $66.60 | $48.93 | $39.75 | $341.98 |
| CLC + DCLC | $0.00 | $104.16 | $93.85 | $91.91 | $83.16 | $373.08 |
| Other costs | $4.91 | $6.16 | $5.09 | $4.03 | $3.42 | $23.61 |
| Total | $199.80 | $286.13 | $241.53 | $207.86 | $178.10 | $1,113.42 |
| **Cost per patient and year, ‘US + AFP’** |  |  |  |  |  |  |
| Surveillance | $62.08 | $52.12 | $42.84 | $34.83 | $28.54 | $220.40 |
| HCC treatment | $27.56 | $39.56 | $30.79 | $26.25 | $21.04 | $145.19 |
| False positives | $107.23 | $89.06 | $74.23 | $56.41 | $48.10 | $375.02 |
| CLC + DCLC | $0.00 | $104.16 | $93.85 | $91.91 | $83.16 | $373.08 |
| Other costs | $4.84 | $6.25 | $5.05 | $4.16 | $3.47 | $23.77 |
| Total | $201.70 | $291.14 | $246.77 | $213.55 | $184.30 | $1,137.46 |

The total cost for the intervention (GAAD or ‘US + AFP) and the comparator (‘US + AFP’ only) was estimated by combining the estimated patient numbers per year (Table S4) with the estimated cost per year (Table S5). The total cost of the intervention included both the cost from new patients screened using GAAD and the cost of the new patients who were screened using ‘US + AFP’. For the comparator, all new patients were screened using ‘US + AFP’. The difference in total cost between the intervention and the comparator is the budget impact, and provide an estimate of how total cost for HCC surveillance in Thailand would be affected from a scenario where all CLC patients are screened using the standard-of-care, to a scenario where some new patients would instead be screened using GAAD. The total cost for both intervention and comparator, as well as the budget impact is shown in Table S6. The estimates indicate the HCC surveillance using GAAD would be associated with a small cost-saving, which would grow over time, for a total saving of $405,931 over 5 years. The main cost-saving from GAAD compared to ‘US + AFP’ is it the avoidance of costs associated false positive diagnoses; by contrast, costs for HCC surveillance and HCC treatment costs would increase slightly when using GAAD.

**S6 Table. Estimated budget impact of switching from routine surveillance using US + AFP to GAAD, per screened individual**

|  | **Year 1** | **Year 2** | **Year 3** | **Year 4** | **Year 5** | **Total** |
| --- | --- | --- | --- | --- | --- | --- |
| **Total cost, intervention (GAAD or ‘US + AFP)** |  |  |  |  |  |  |
| Surveillance | $1,431,724 | $2,579,136 | $3,481,487 | $4,184,497 | $4,737,737 | $16,414,581 |
| HCC treatment | $636,528 | $1,500,349 | $2,141,837 | $2,659,688 | $3,055,056 | $9,993,458 |
| False positives | $2,449,491 | $4,360,357 | $5,847,845 | $6,897,291 | $7,725,618 | $27,280,602 |
| CLC + DCLC | $0 | $2,275,473 | $4,223,214 | $6,035,170 | $7,592,631 | $20,126,488 |
| Other costs | $111,598 | $247,857 | $352,833 | $434,368 | $498,916 | $1,645,573 |
| **Total** | **$4,629,340** | **$10,963,173** | **$16,047,216** | **$20,211,014** | **$23,609,958** | **$75,460,702** |
| **Total cost, comparator (‘US + AFP only)** |  |  |  |  |  |  |
| Surveillance | $1,427,486 | $2,566,078 | $3,455,086 | $4,141,677 | $4,676,278 | $16,266,605 |
| HCC treatment | $633,627 | $1,497,723 | $2,136,699 | $2,654,226 | $3,048,300 | $9,970,576 |
| False positives | $2,465,696 | $4,411,178 | $5,951,707 | $7,063,776 | $7,964,616 | $27,856,974 |
| CLC + DCLC | $0 | $2,275,473 | $4,223,214 | $6,035,170 | $7,592,631 | $20,126,488 |
| Other costs | $111,287 | $247,752 | $352,614 | $434,703 | $499,635 | $1,645,991 |
| **Total** | **$4,638,096** | **$10,998,203** | **$16,119,320** | **$20,329,552** | **$23,781,461** | **$75,866,633** |

|  | **Year 1** | **Year 2** | **Year 3** | **Year 4** | **Year 5** | **Total** |
| --- | --- | --- | --- | --- | --- | --- |
| **Budget impact** |  |  |  |  |  |  |
| Surveillance | $4,238 | $13,058 | $26,401 | $42,820 | $61,458 | $147,976 |
| HCC treatment | $2,901 | $2,627 | $5,138 | $5,462 | $6,756 | $22,883 |
| False positives | -$16,205 | -$50,820 | -$103,862 | -$166,485 | -$238,998 | -$576,371 |
| CLC + DCLC | $0 | $0 | $0 | $0 | $0 | $0 |
| Other costs | $311 | $106 | $219 | -$335 | -$719 | -$418 |
| **Total** | **-$8,756** | **-$35,030** | **-$72,104** | **-$118,538** | **-$171,503** | **-$405,931** |

Note: All costs in USD. Budget impact results are based upon a different random patient draw than the cost-effectiveness analysis, hence small cost discrepancies between the same surveillance methods in the two different analyses are expected.
