# Supplementary material for: Economic evaluation of biomarker-based surveillance for Hepatocellular Carcinoma in Thai patients with Compensated Liver Cirrhosis
Source: PLoS One. 2026 Jan 5;21(1):e0337913. doi: 10.1371/journal.pone.0337913 (PMC12768342; doi:10.1371/journal.pone.0337913)
Supplement: S4 Appendix — (DOCX) [file pone.0337913.s004.docx]

# Appendix 4: Comparison of GAAD vs GALAD

In the base case analysis, screening using GALAD resulted in minor health improvement compared to GAAD, but its substantially higher costs made this option economically unjustifiable. These higher costs mainly stemmed from two factors. First, the higher false positive detection rate of GALAD (stemming from its lower specificity in early-stage HCC) necessitated more unnecessary confirmatory tests. Second, the higher price of GALAD substantially raised its surveillance costs. Since the price of alternative biomarker-based screening methods may differ between providers, an additional scenario analysis was performed to assess how results may change if alternative GALAD prices were used.

A probabilistic analysis was performed using 100,000 microsimulations with price parity between GAAD and GALAD surveillance costs. Results (presented below in Fig S2) suggested that GAAD was likely to be the preferred biomarker-based approach from a cost-effectiveness perspective even at identical screening costs. In all simulations, total costs remained higher for GALAD than GAAD due to its higher associated costs of false positive diagnoses. At a willingness-to-pay (WTP) threshold of $4,800, GALAD was cost-effective versus GAAD in less than 3% of simulations, though the probability could increase with higher WTP thresholds. This supplementary analysis confirmed that amongst the compared biomarker-based approaches for routine HCC surveillance, GAAD is likely to be the most cost-effective.

**S2 Fig. Cost-effectiveness analysis of GAAD versus GALAD as the screening method for routine HCC surveillance among CLC patients when screening costs are at parity**


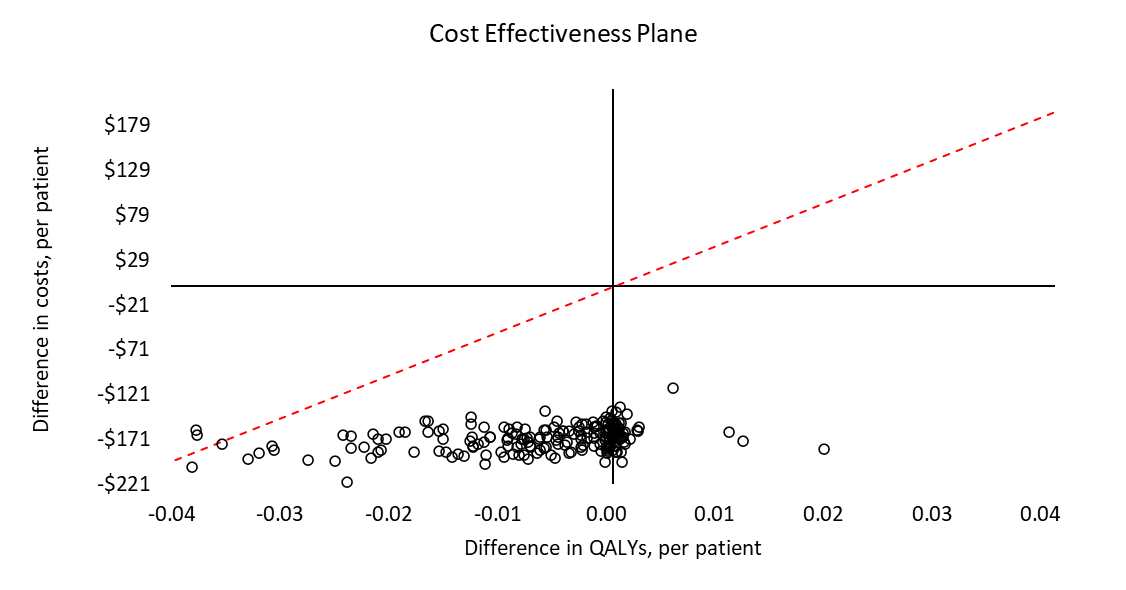


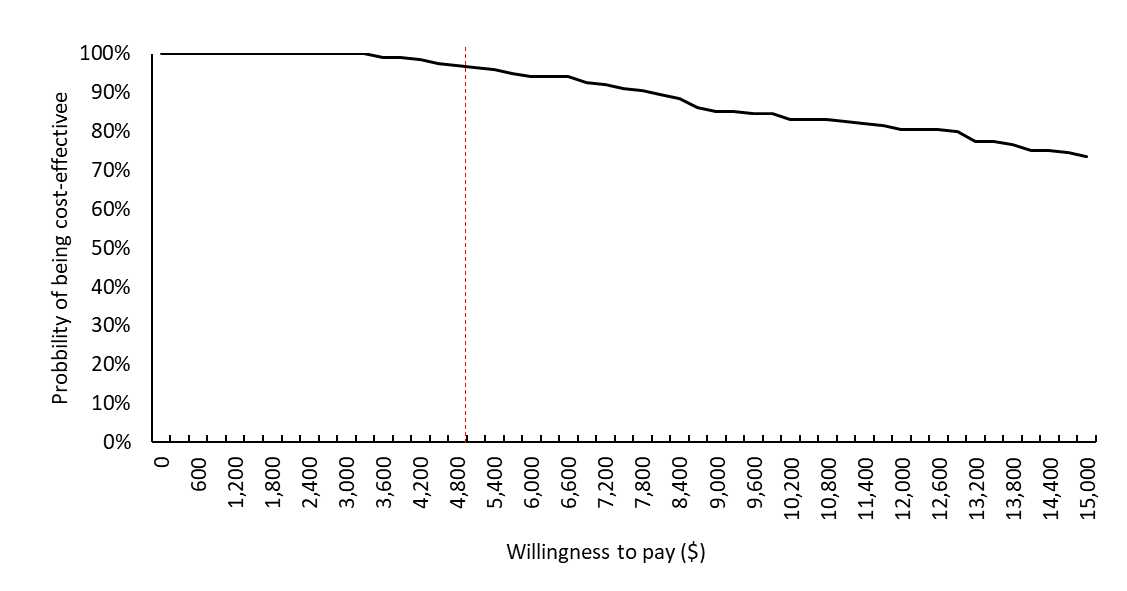


Top panel: Cost-effectiveness plane for GAAD versus GALAD; negative costs means that GAAD is cost-saving
Bottom panel: Cost-effectiveness acceptability curve showing the likelihood that GAAD is cost-effective versus GALAD at alternative willingness-to-pay thresholds
Abbreviations: CLC: compensated liver cirrhosis, HCC: hepatocellular carcinoma, QALY: quality-adjusted life year

# References

1. Sangmala P, Chaikledkaew U, Tanwandee T, Pongchareonsuk P. Economic evaluation and budget impact analysis of the surveillance program for hepatocellular carcinoma in thai chronic hepatitis B patients. Asian Pacific journal of cancer prevention: APJCP. 2014;15(20):8993-9004.

2. Riewpaiboon A. Standard cost lists for health economic evaluation in Thailand. J Med Assoc Thai. 2014;97 Suppl 5:S127-34.

3. Thongsawat S, Piratvisuth T, Pramoolsinsap C, Chutaputti A, Tanwandee T, Thongsuk D. Resource Utilization and Direct Medical Costs of Chronic Hepatitis C in Thailand: A Heavy but Manageable Economic Burden. Value in Health Regional Issues. 2014;3:12-8.

4. Chanree P, Siripongsakun S, Sangmala P, Hiranrat P, Chotipanich C. Cost Effectiveness Analysis of Hepatocellular Carcinoma Surveillance using Abdominal Ultrasound in Hepatitis B Patients. RSU International Research Conference 2022.

5. Liver Association of T. แนวทางการดูแลมะเร็งตับในประเทศไทย-พศ [Thailand Guideline for Management of Hepatocellular Carcinoma 2021 (Revised Edition)]. 2021.

6. Poovorawan K, Treeprasertsuk S, Thepsuthammarat K, Wilairatana P, Kitsahawong B, Phaosawasdi K. The burden of cirrhosis and impact of universal coverage public health care system in Thailand: Nationwide study. Ann Hepatol. 2015;14(6):862-8.

7. Chitapanarux T, Phornphutkul K. Risk Factors for the Development of Hepatocellular Carcinoma in Thailand. J Clin Transl Hepatol. 2015;3(3):182-8.

8. Fleming KM, Aithal GP, Card TR, West J. The rate of decompensation and clinical progression of disease in people with cirrhosis: a cohort study. Aliment Pharmacol Ther. 2010;32(11-12):1343-50.

9. Kondo T, Koroki K, Kanzaki H, Kobayashi K, Kiyono S, Nakamura M, et al. Impact of acute decompensation on the prognosis of patients with hepatocellular carcinoma. PLOS ONE. 2022;17(1):e0261619.

10. Kitiyakara T, Leerapun A, Sutthivanan C, Poovorawan K, Pan-Ngum W, Soonthornworasiri N, et al. Regional Differences in Admissions and Treatment Outcomes for Hepatocellular Carcinoma Patients in Thailand. Asian Pacific Journal of Cancer Prevention: APJCP. 2022;23(11):3701.

11. Zhang M, Li Y, Fan Z, Shen D, Huang X, Yu Q, et al. Assessing health-related quality of life and health utilities in patients with chronic hepatitis B-related diseases in China: a cross-sectional study. BMJ Open. 2021;11(9):e047475.

12. Lima PH, Fan B, Bérubé J, Cerny M, Olivié D, Giard JM, et al. Cost-Utility Analysis of Imaging for Surveillance and Diagnosis of Hepatocellular Carcinoma. AJR Am J Roentgenol. 2019;213(1):17-25.

13. Reig M, Forner A, Rimola J, Ferrer-Fàbrega J, Burrel M, Garcia-Criado Á, et al. BCLC strategy for prognosis prediction and treatment recommendation: The 2022 update. J Hepatol. 2022;76(3):681-93.

14. Sethasine S, Simasingha N, Ratana-Amornpin S, Mahachai V. Real world for management of hepatocellular carcinoma: a large population-based study. Scandinavian Journal of Gastroenterology. 2023:1-6.

15. Decharatanachart P, Pan-Ngum W, Peeraphatdit T, Tanpowpong N, Tangkijvanich P, Treeprasertsuk S, et al. Cost-Utility Analysis of Non-Contrast Abbreviated Magnetic Resonance Imaging for Hepatocellular Carcinoma Surveillance in Cirrhosis. Gut Liver. 2024;18(1):135-46.

16. Rattanasupar A, Chartleeraha S, Akarapatima K, Chang A. Factors that Affect the Surveillance and Late-Stage Detection of a Newly Diagnosed Hepatocellular Carcinoma. Asian Pac J Cancer Prev. 2021;22(10):3293-8.

17. Wong MCS, Huang JLW, George J, Huang J, Leung C, Eslam M, et al. The changing epidemiology of liver diseases in the Asia–Pacific region. Nature Reviews Gastroenterology & Hepatology. 2019;16(1):57-73.

18. World Health Organization Thailand Population 2023.
